# Supplementary material for: Single-Ion Magnetism of the [DyIII(hfac)4]− Anions in the Crystalline Semiconductor {TSeT1.5}●+[DyIII(hfac)4]− Containing Weakly Dimerized Stacks of Tetraselenatetracene
Source: Int J Mol Sci. 2024 Jul 24;25(15):8068. doi: 10.3390/ijms25158068 (PMC11311655; doi:10.3390/ijms25158068)
Supplement: Supplementary file 1 [file ijms-25-08068-s001.zip › ijms-3092624-supplementary.pdf]

Supplementary Information for the manuscript:

Single-Ion Magnetism of the $[Dy^{III}(hfac)_4]^-$ Anions in the Crystalline Semiconductor $\{TSeT_{1.5}\}^{\bullet+}[Dy^{III}(hfac)_4]^-$ Containing Weakly Dimerized Stacks of Tetraselenatetracene

Alexandra M. Flakina,[a] Dmitry I. Nazarov,[a] Maxim A. Faraonov,[a] Ilya A. Yakushev,[b] Aleksey V. Kuzmin,[c] Salavat S. Khasanov,[c] Vladimir N. Zverev,[c] Akihiro Otsuka,[d] Hideki Yamochi,[d] Hiroshi Kitagawa[d] and Dmitri V. Konarev*[a]

[a]Federal Research Center of Problems of Chemical Physics and Medicinal Chemistry RAS, Chernogolovka, Moscow region, 142432 Russia; E-mail: konarev3@yandex.ru;

[b]Kurnakov Institute of General and Inorganic Chemistry, Russian Academy of Sciences, Moscow, 119991 Russia;

[c]Institute of Solid State Physics RAS, Chernogolovka, Moscow region, 142432 Russia;

[d]Division of Chemistry, Graduate School of Science, Kyoto University, Sakyo-ku, Kyoto 606-8502, Japan.

Optical properties

Table S1. IR-spectra (cm^{-1}) of starting compounds and salt **1** measured in KBr pellets.

Components	TSeT	$Dy^{III}(hfac)_3$	Salt **1**
TSeT	458s		458w
	593w		585w*
	627m		627w
	658 w		660m*
	680w		693w
	736vs		742w
	770m		-
	797 w		796m
	867 s		871m* 881m* sp
	937 ms		922w
	955 m		950m 957 m sp
	1034 m		1042w
	1121 m		-
	1147 w		1144 vs
	1183 w		-
	1258s		1254s*
	1273s		-
	1293vs		-
	1344 m		1357m
	1382 m		-
	1430 (sp)		-
	1455s		1456s*
	1478m		-
	1514m		1505m 1523m sp
	1560 m		1556w*
	1610 m		1605w
	2851 w		2850w
	2972 w		2970w
		417w	418w
$Dy^{III}(hfac)_3$		445w	436w
		465w	471w
		527w	527w
		584m	586w*
		660s	660m*
		732w	-
		802m 809m sp	-
		872w	871m* 881m* sp
		1096s	1098w
		1134s	1144vs
		1196s	1193s
		1254m	1254s*
		1452s	1456m*
		1482w	-
		1540w	1556w*
		1566w	-
		1623m	1605w
		1646m	1651s
		3154w	3154w

Abbreviation: w: weak, m: middle, s- strong, vs-very strong,

sp. – split band, *: bands are overlapped

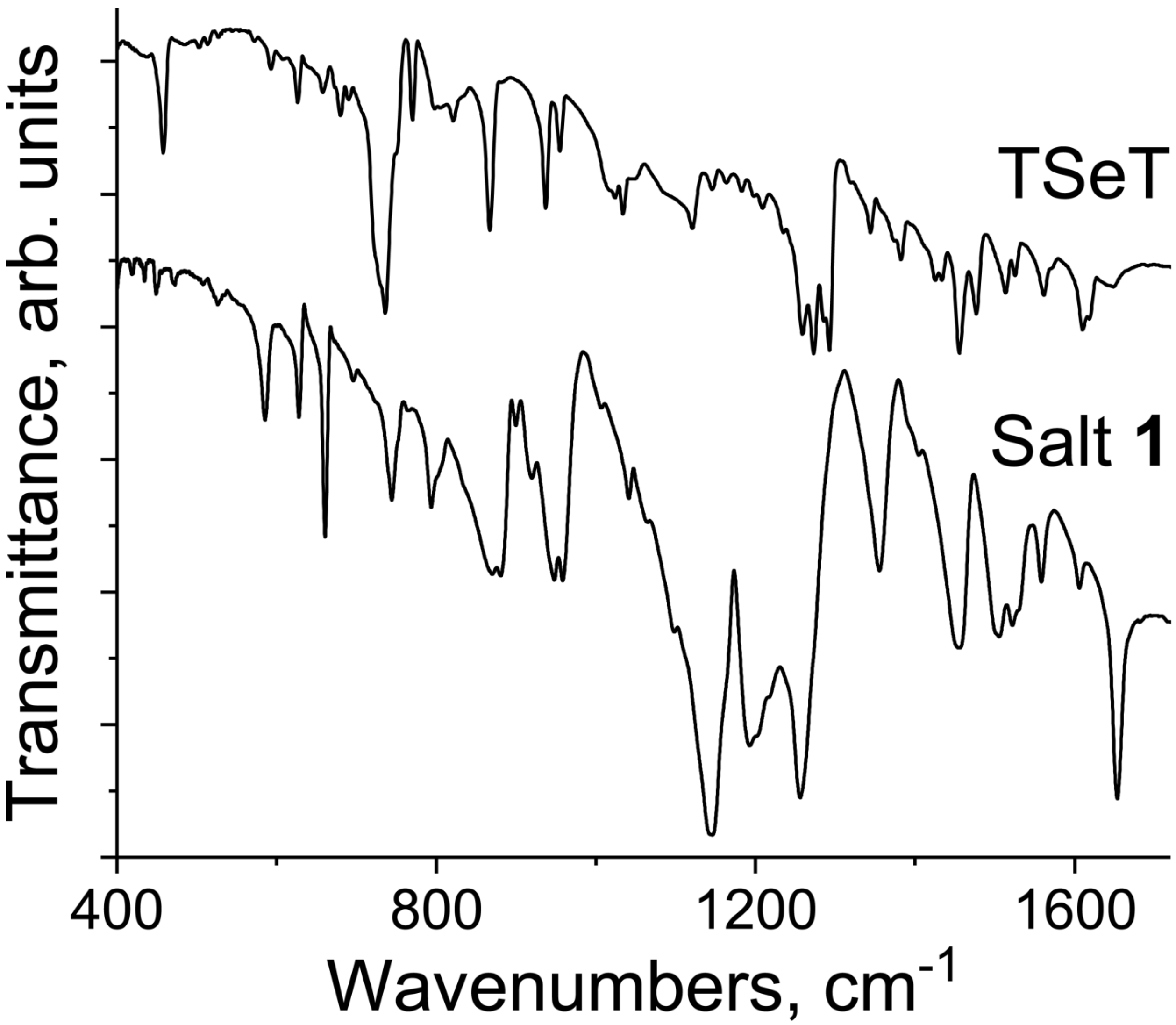

Figure S1. IR spectra of starting TSeT and $(TSeT)_{1.5}^{\bullet+}\{Dy^{III}(hfac)_4\}^-$ (**1**) in KBr pellets prepared in anaerobic conditions.

Figure S1 shows IR-spectrum of $(TSeT)_{1.5}^{\bullet+}\{Dy^{III}(hfac)_4\}^-$ (**1**), and the spectrum of starting TSeT is shown for comparison. It is seen that the spectrum of **1** is a superposition of the absorption bands of TSeT and the $[Dy^{III}(hfac)_4]^-$ anion. This makes it difficult to decipher and assign vibration bands (Table S1). The band of carbon skeleton vibrations in TSeT at 1610 cm^{-1} is shifted by 5 cm^{-1} toward lower frequencies. Previously, a similar shift by about 8 cm^{-1} was observed in the iodine salts of TSeT and TTT (tetrathiatetracene) [45-47]. The spectrum of **1** also contains bands at 871, 881, 950, 957, 1217, 1254, 1357, and 1456 cm^{-1} which can be attributed to TSeT (Figure S1). The same bands at 1460 and 1356 cm^{-1} were observed in the spectra of TTT and TSeT iodides, in which they were attributed to the $TTT^{\bullet+}$ and $TSeT^{\bullet+}$ cations [45, 46]. The spectra of $TSeTI_x$ also show a band at 888 cm^{-1} [46] arising from deformational CH vibrations, and this band is manifested in the spectrum of **1** as a split band at 871 and 881 cm^{-1} shifted essentially in comparison with the spectrum of starting TSeT (867 cm^{-1}). The bands at 1217 and 1254 cm^{-1} in the spectrum of **1** can be attributed to vibrations of C-Se bonds located at 1244 and 1293 cm^{-1} in the spectrum of starting TSeT.

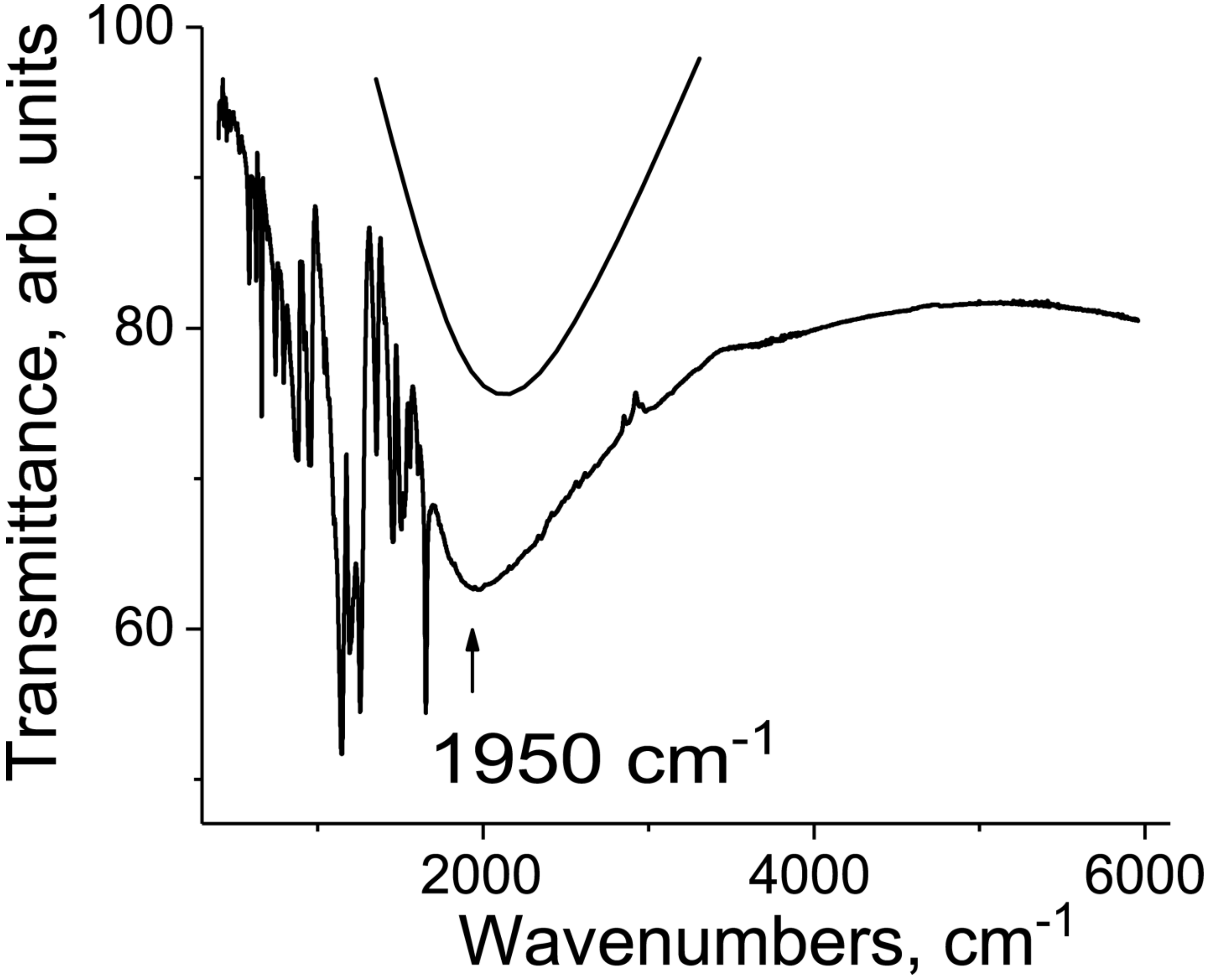

Figure S2. IR spectrum of $(TSeT)_{1.5}^{\bullet+}\{Dy^{III}(hfac)_4\}^-$ (**1**) showing the low-energy charge transfer band measured in KBr pellet prepared in anaerobic conditions.

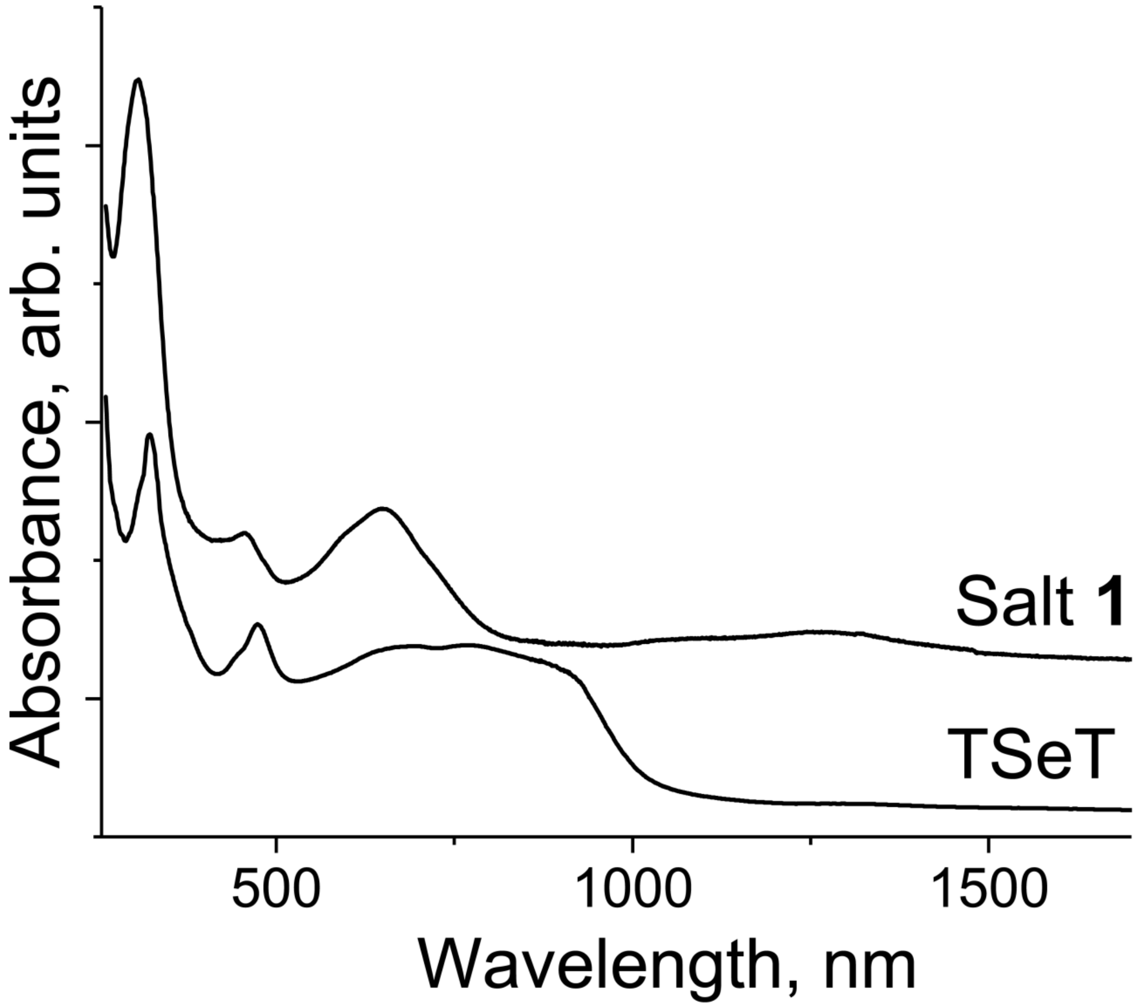

Figure S3. Spectra of starting TSeT and salt $(TSeT)_{1.5}^{\bullet+}\{Dy^{III}(hfac)_4\}^-$ (**1**) in the 260-1700 nm range in KBr pellet prepared in anaerobic conditions.

Table S2. UV-visible-NIR spectra of TSeT and salt $(TSeT)_{1.5}^{\bullet+}\{Dy^{III}(hfac)_4\}^-$ (**1**).

Compound	Bands in the UV-visible range, nm	Bands in the NIR range, nm
TSeT	318, 480, 602, 761	
**1**	307, 452, 650	1260, 5100 (1950 cm^{-1})

Previously, electronic absorption spectra of various TSeT complexes were analyzed [46]. The spectra showed bands for neutral $TSeT^{0}$ at 319, 468, and 704 nm, and a series of additional bands assigned to the $TSeT^{\bullet+\cdot}$ radical cation in $TSeTI_x$ was found at 341, 390, 642, 1088, and 1280 nm [46]. The spectrum of **1** manifests bands at 307, 452, 650, 1260 nm (Figure S3), which can be attributed to the TSeT species having charge closer to +1 and 0. An interesting feature of **1** is the presence of very low-energy absorption with maximum at 1950 cm^{-1} or 5100 nm (Figure S2). Most probably it can be attributed to charge transfer between TSeT species involved in the dimers or between TSeT from the dimers and monomers. Previously, wide absorption with maxima at 4000-5000-cm^{-1} was also observed in the salts containing TSeT stacks, and this absorption was attributed to electron transfer between $TSeT^{\bullet+\cdot}$ and $TSeT^{0}$.[46]

Crystal structure

Table S3. Shape analysis of salt **1** using SHAPE 2.1 software. [32, 33] The smaller value corresponds to geometry of the coordination polyhedron of a metal center closer to the perfect one.

N	Abbreviation	Coordination polyhedron	Symmetry	**100 K**
1	OP-8	Octagon	D_{8h}	33.440
2	HPY-8	Heptagonal pyramid	C_{7v}	24.570
**3**	HBPY-8	Hexagonal bipyramid	D_{6h}	12.706
4	CU-8	Cube	O_h	5.265
5	CU-8	Square antiprism	D_{4d}	2.855
6	TDD-8	Triangular dodecahedron	D_{2d}	0.374
7	JGBF-8	Johnson - Gyrobifastigium (J26)	D_{2d}	15.915
8	JETBPY-8	Johnson - Elongated triangular bipyramid (J14)	D_{3h}	28.025
9	JBTP-8	Johnson - Biaugmented trigonal prism (J50)	C_{2v}	3.572
10	BTPR-8	Biaugmented trigonal prism	C_{2v}	3.035
11	JSD-8	Snub disphenoid (J84)	D_{2d}	3.789
12	TT-8	Triakis tetrahedron	T_d	6.123
13	ETBPY-8	Elongated trigonal bipyramid	D_{3h}	25.479

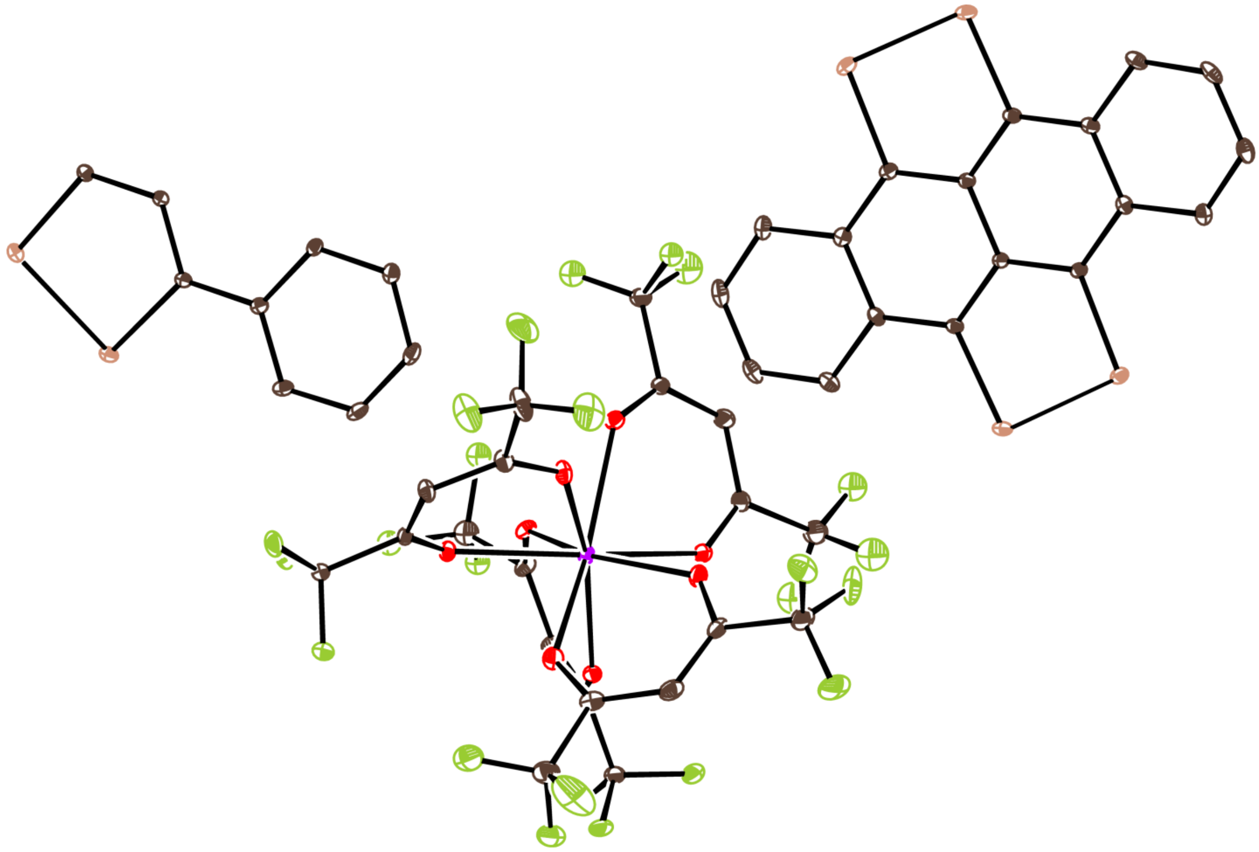

Figure S4. Crystallographically independent units in the structure of **1**: one and half independent TSeT molecules and one $\{Dy^{III}(hfac)_4\}^-$ anion. Disorder in some of CF_3 groups of the anion (disorder between two orientations) is not shown. Carbon is brown, fluorine is green, oxygen is red, and dysprosium is violet. Ellipsoid probability is 25%.

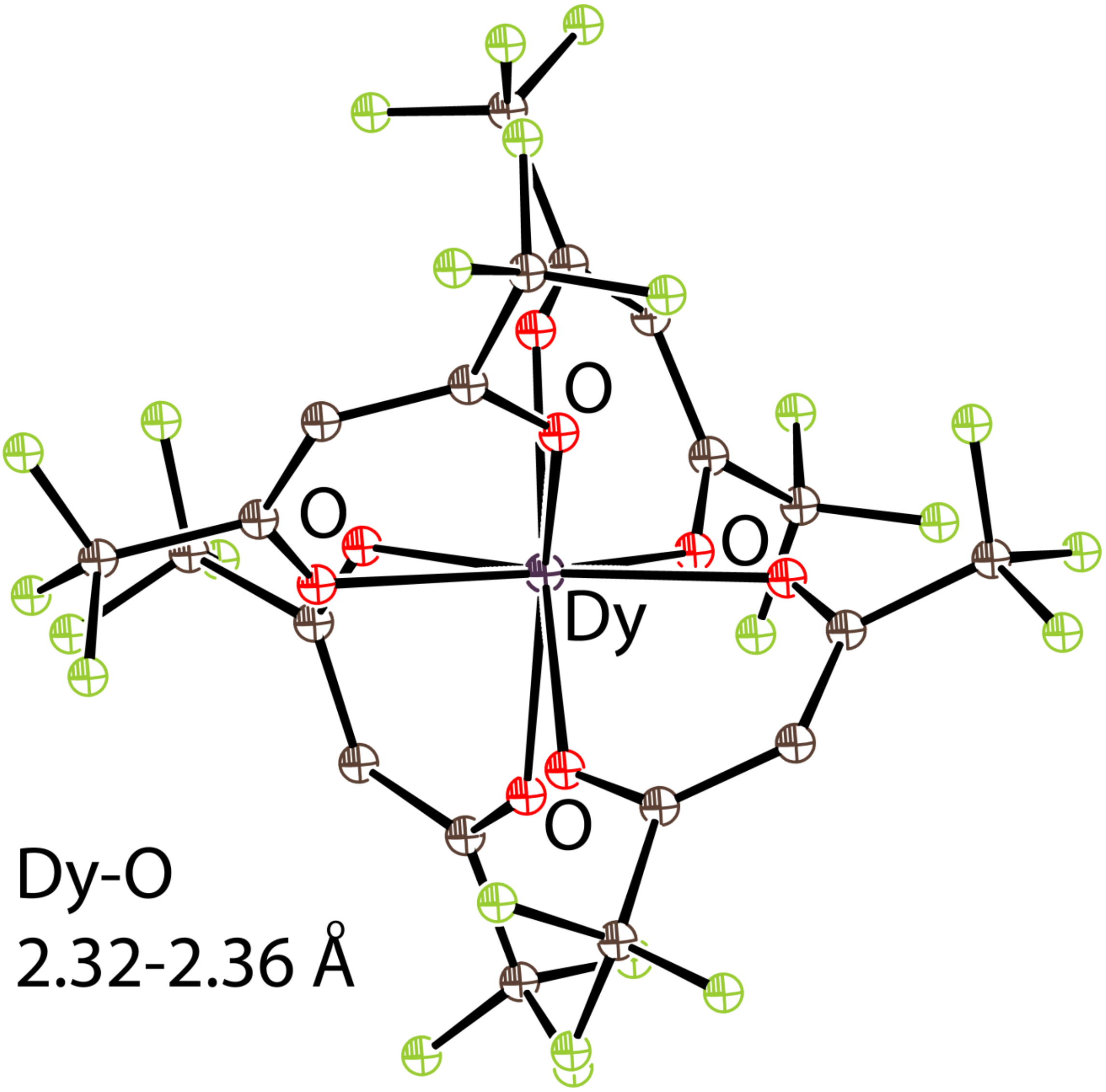

Figure S5. View on the geometry the $\{Dy^{III}(hfac)_4\}^-$ anion in salt **1**. Carbon is brown, fluorine is green, oxygen is red, selenium is pink and dysprosium is violet.

Resistivity measurements on oriented single crystal by four-probe technique.

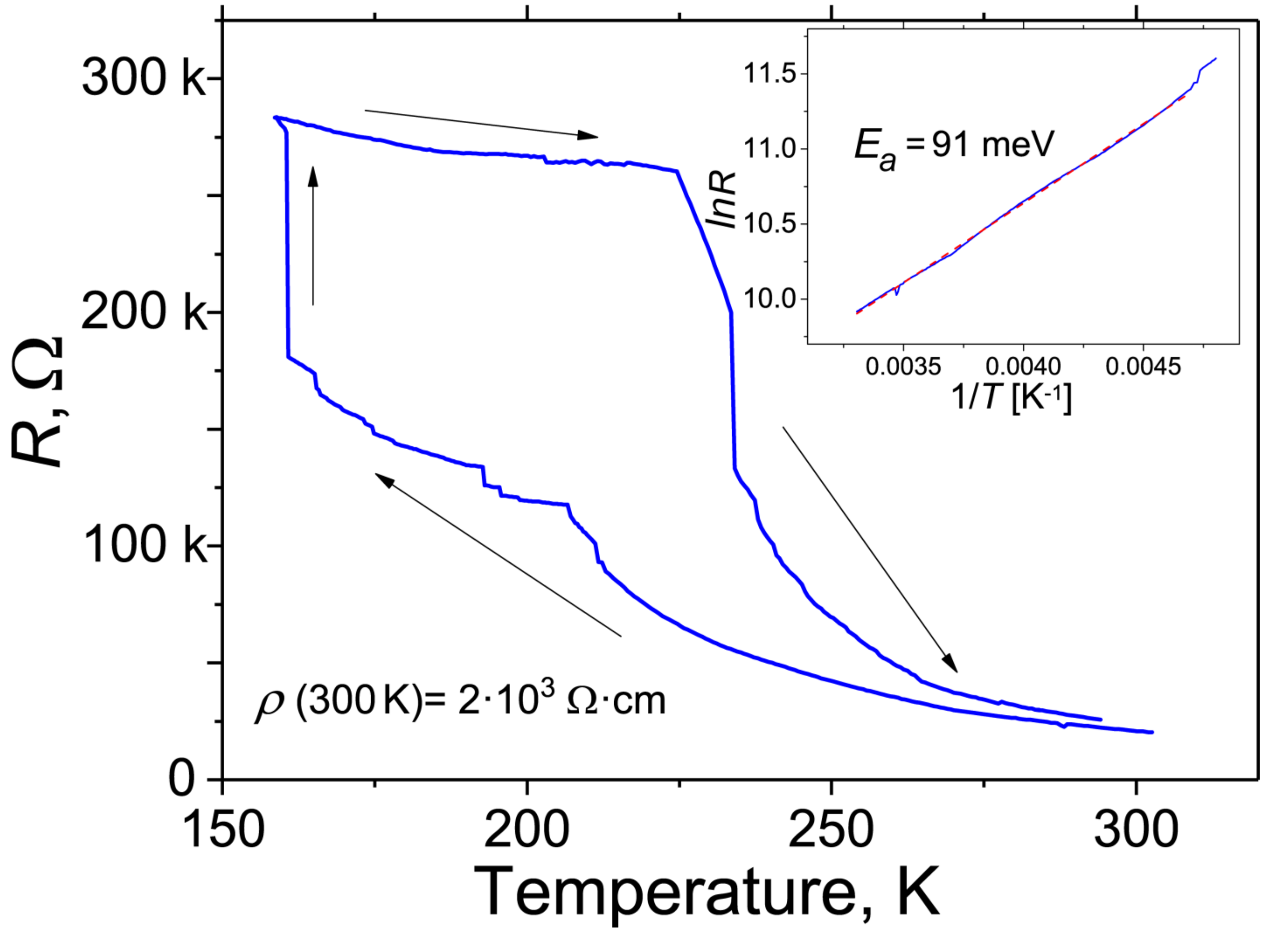

Figure S6. Resistivity measurements on single crystal by four-probe technique. The direction of the resistivity measurements on the single crystal was confirmed by X-ray diffraction in such a way that measurement was made along the crystallographic *a* axis and the stacks of TSeT molecules. Inset shows determination of activation energy of 91 meV (at 300-210 K) for semiconducting behavior of **1**.

Fitting of magnetic data by PHI.

The spin Hamiltonian for the fitting of the susceptibility data of the complex **1** in PHI was used as

$$\hat{\mathcal{H}}_{\mathrm{ZEE}} = \mu_B \hat{\boldsymbol{S}} \cdot g_J \cdot \hat{\boldsymbol{B}} \qquad (1)$$

$$\hat{\mathcal{H}}_{\mathrm{ZFS}} = D\left\{\hat{\boldsymbol{S}}_z^2 - \frac{1}{3}S(S+1)\right\} \qquad (2)$$

where ***S*** and ***B*** with hats, μ_B, g_J, *D*, and *S* refer to operators of spin, magnetic field, the Bohr magneton, *g*-factor for lanthanide, axial zero-field splitting (ZFS) constant, and total spin on the metal ion, respectively. The Dy(III) ion with ground state free ion term symbol $^6H_{15/2}$ was considered.

Magnetic properties

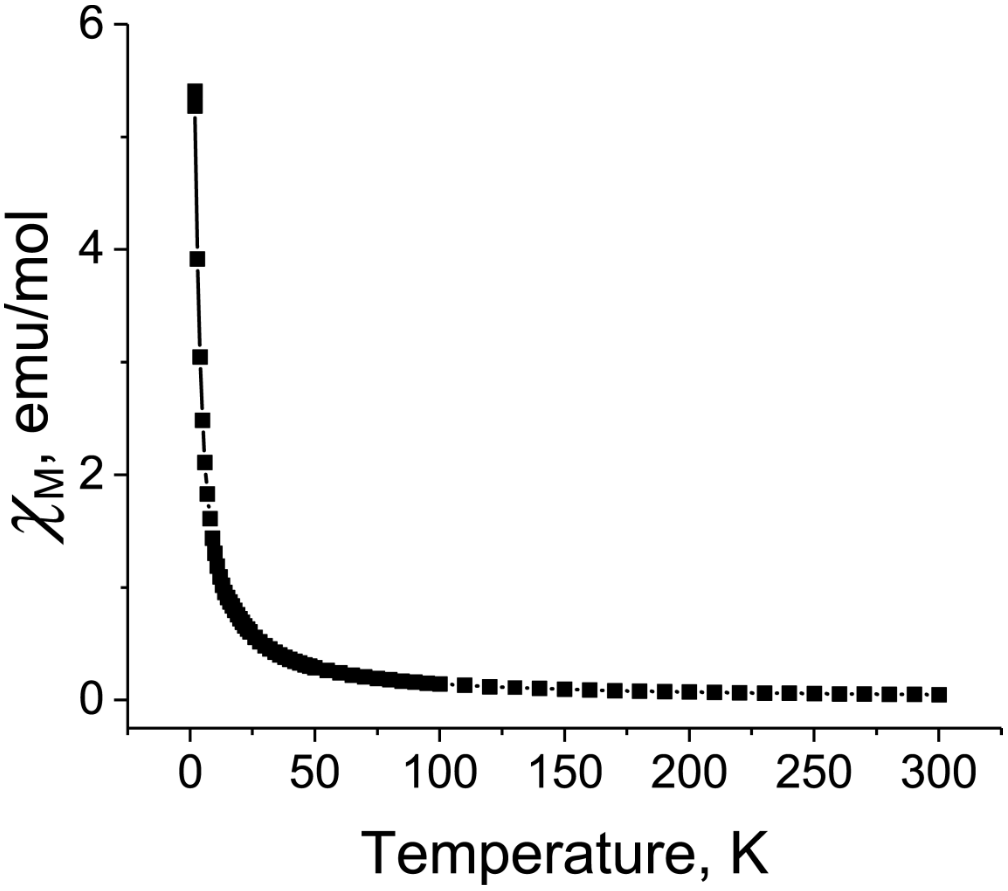

Figure S7. Data of SQUID measurements for polycrystalline **1**. The temperature dependence of molar magnetic susceptibility is shown in the 1.9-300 K range after the subtraction of a temperature independent contribution. ZFC curve is shown.

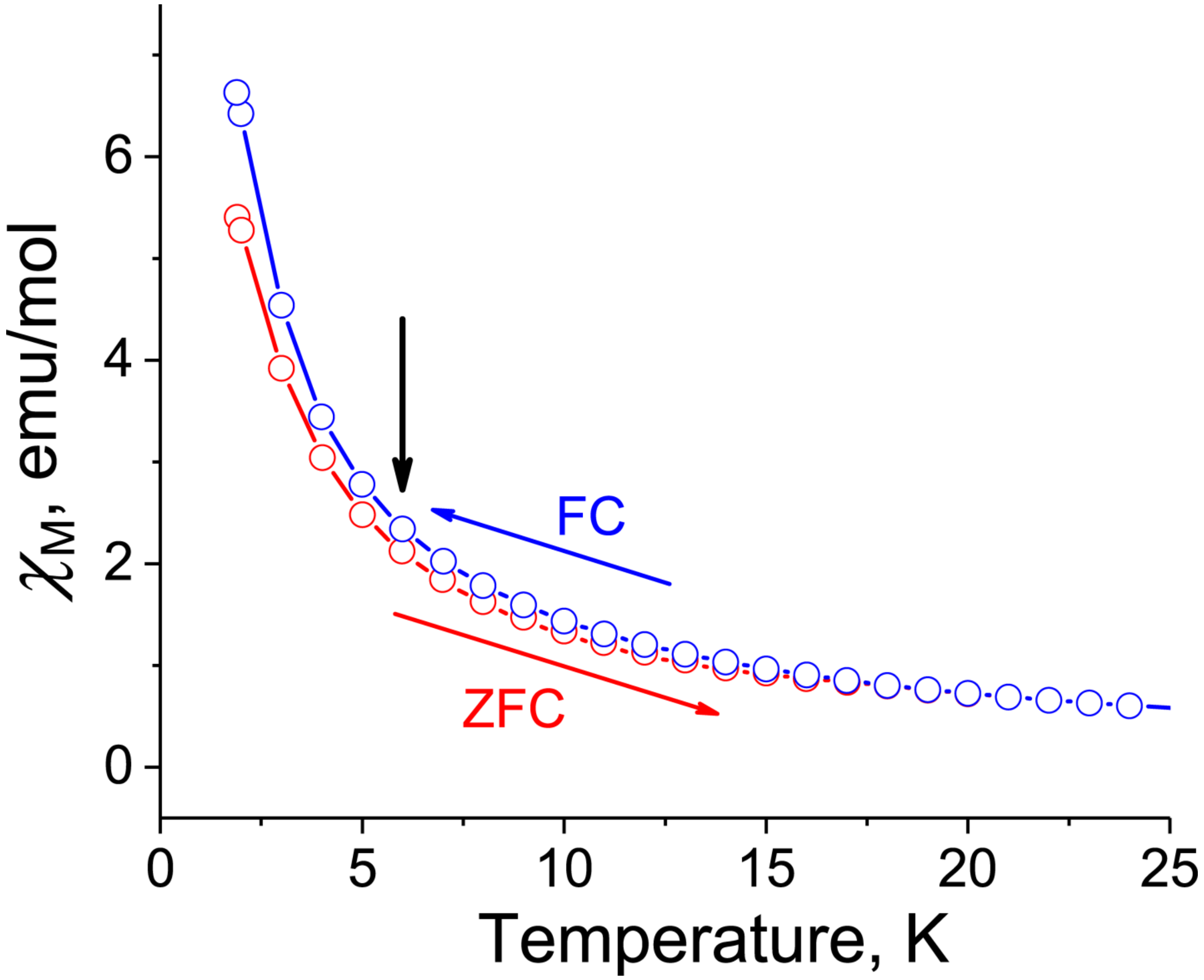

Figure S8. Temperature dependence of molar magnetic susceptibility measured in zero-field cooling (ZFC) and field-cooling (1000 Oe, FC) conditions. In spite of that small discrepancy between the curves is observed below 15 K, a noticeable difference between them can be found only below 8-5 K.

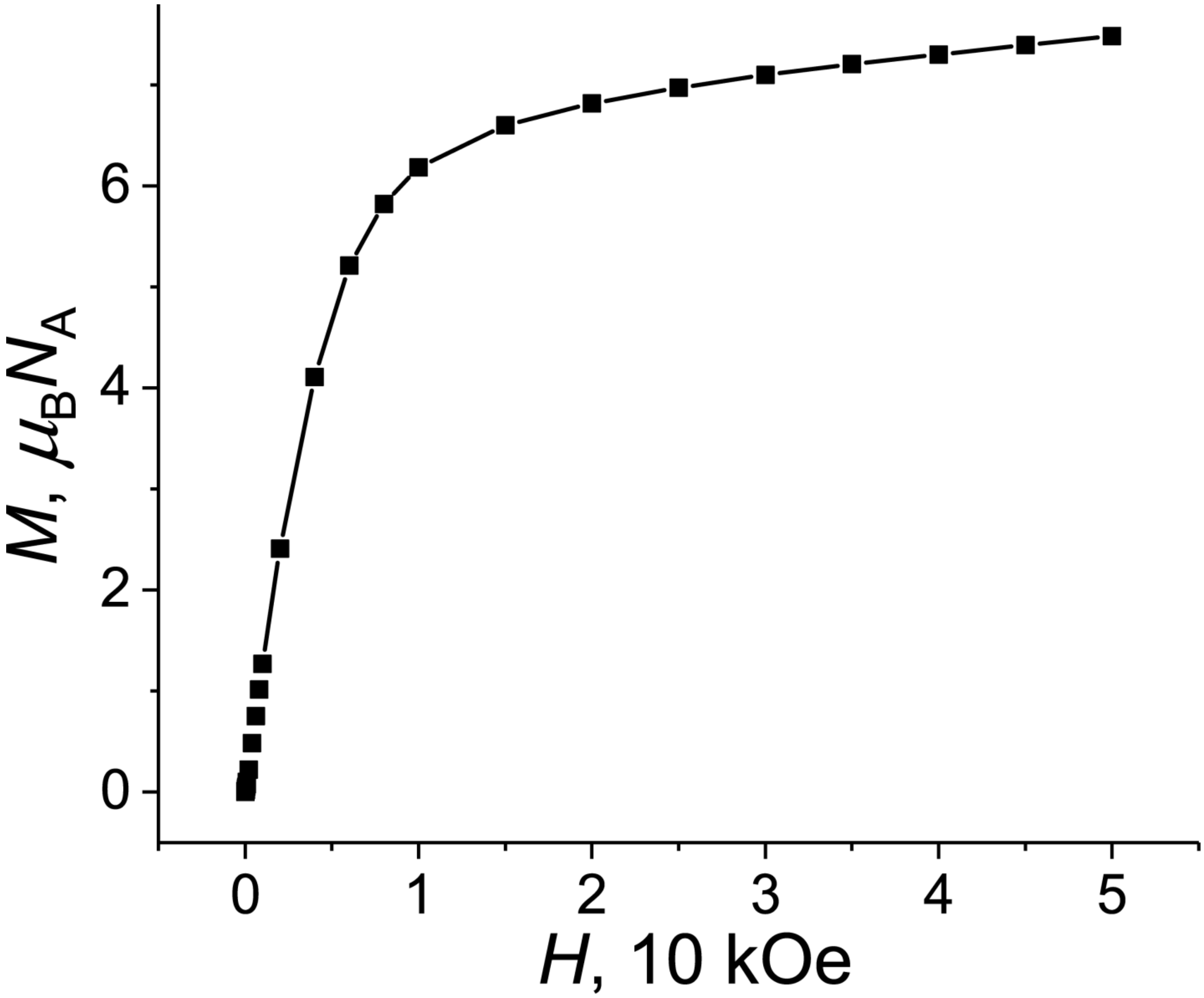

Figure S9. Dependence of magnetization of polycrystalline **1** *vs* magnetic field (black circles) up to 50 kOe magnetic field at 2 K.

.

AC measurements

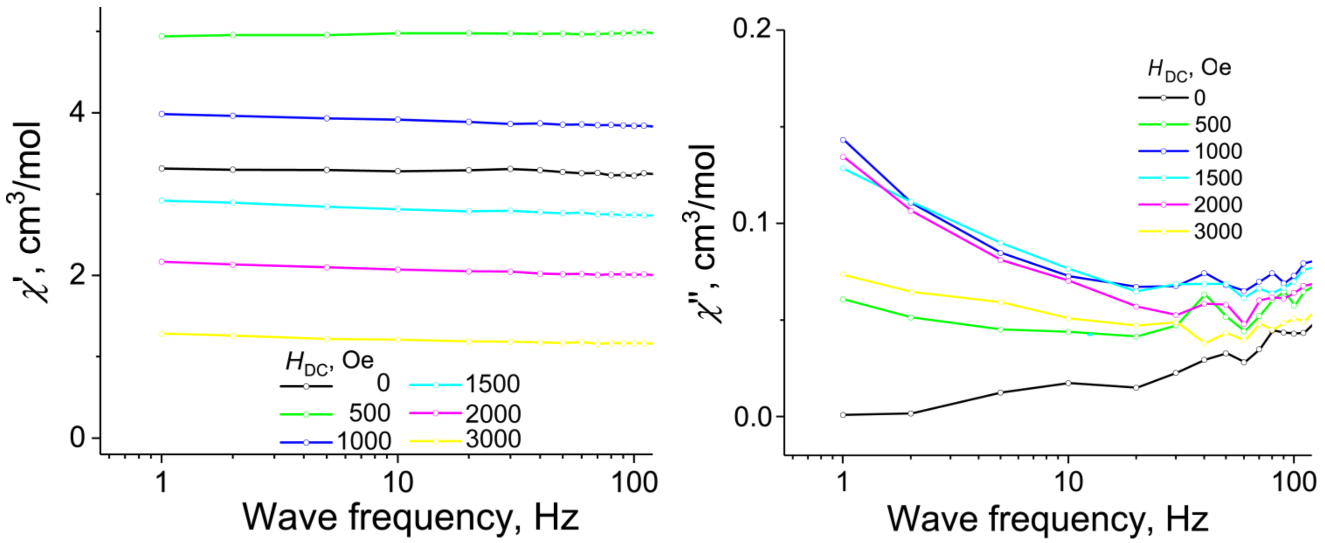

Figure S10. Frequency dependences of in-phase (χ') (a) and out-of-phase (χ'') (b) AC susceptibility for **1** at different applied static magnetic fields H_{DC} from 0 to 3000 Oe. Maximum for χ'' is at 1000 Oe (blue curve).

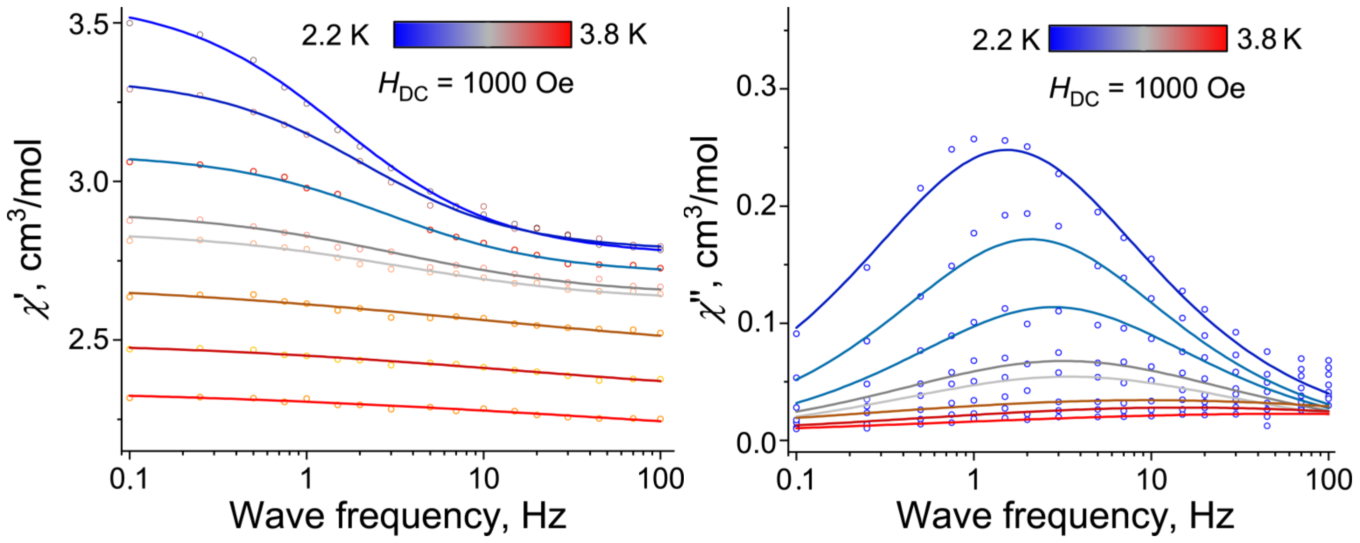

Figure S11. Frequency dependences of in-phase (χ') (a) and out-of-phase (χ'') (b) AC magnetic susceptibilities for **1** in 1000 Oe static magnetic field at 2.2 – 3.8 K. Solid lines represent the fitting of the data by the generalized Debye model.

EPR measurements

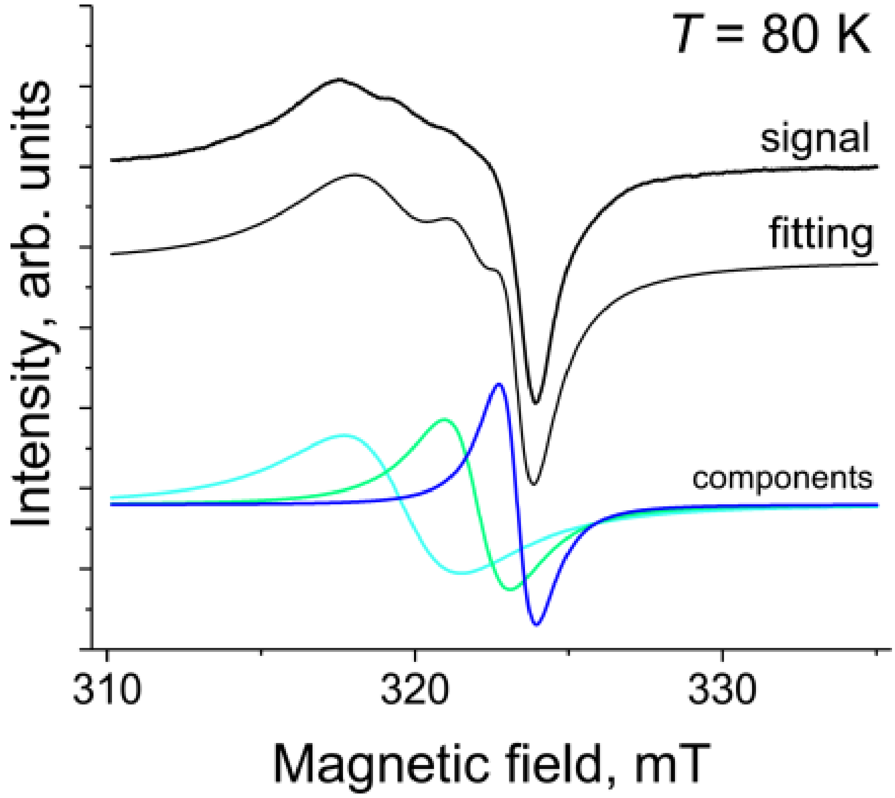

Figure S12. EPR spectrum of polycrystalline **1** at 80 K and fitting of the signal by three Lorentzian lines.

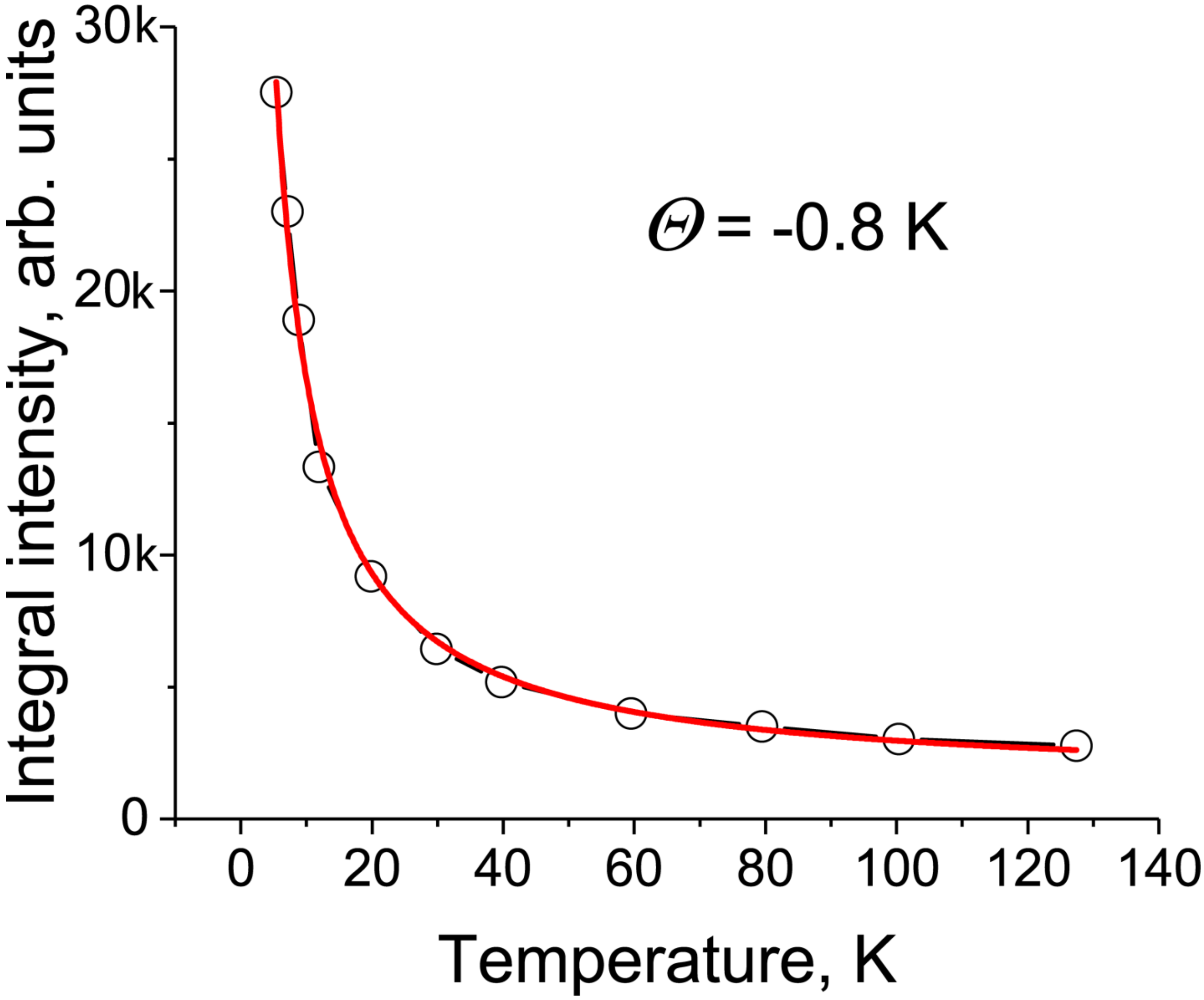

Figure S13. Fitting of integral intensity of the EPR signal from the TSeT monomers in the 5.4-128 K range (all components are taken into account) and fitting of this dependence by the Curie-Weiss law with Weiss temperature of -0.8 K (red curve).

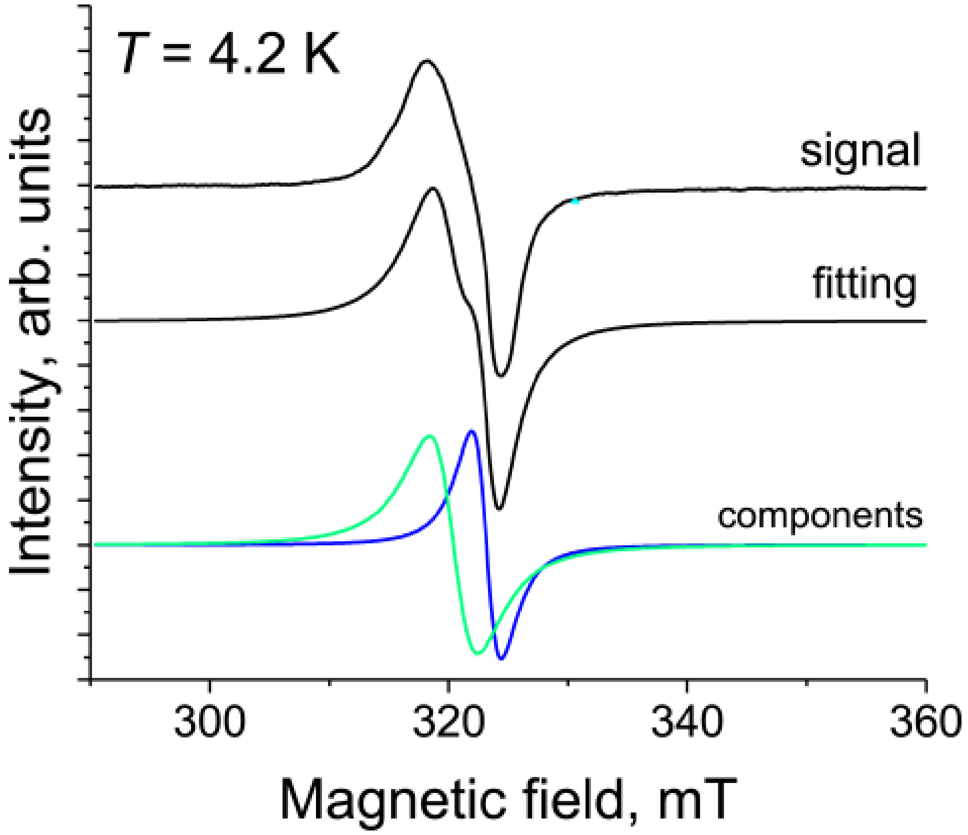

Figure S14. EPR spectrum of polycrystalline **1** at 4.2 K, fitting of the signal by two Lorentzian lines.
